# Elastase-Induced Parenchymal Disruption and Airway Hyper Responsiveness in Mouse Precision Cut Lung Slices: Toward an *Ex vivo* COPD Model

**DOI:** 10.3389/fphys.2016.00657

**Published:** 2017-01-04

**Authors:** Eline M. Van Dijk, Sule Culha, Mark H. Menzen, Cécile M. Bidan, Reinoud Gosens

**Affiliations:** ^1^Department of Molecular Pharmacology, University of GroningenGroningen, Netherlands; ^2^Groningen Research Institute for Asthma and COPD, University Medical Center Groningen, University of GroningenGroningen, Netherlands; ^3^Université Grenoble Alpes, Centre National de la Recherche Scientifique, LIPhyGrenoble, France

**Keywords:** airway mechanics, extracellular matrix, chronic obstructive pulmonary disease

## Abstract

**Background:** COPD is a progressive lung disease characterized by emphysema and enhanced bronchoconstriction. Current treatments focused on bronchodilation can delay disease progression to some extent, but recovery or normalization of loss of lung function is impossible. Therefore, novel therapeutic targets are needed. The importance of the parenchyma in airway narrowing is increasingly recognized. In COPD, the parenchyma and extracellular matrix are altered, possibly affecting airway mechanics and enhancing bronchoconstriction. Our aim was to set up a comprehensive *ex vivo* Precision Cut Lung Slice (PCLS) model with a pathophysiology resembling that of COPD and integrate multiple readouts in order to study the relationship between parenchyma, airway functionality, and lung repair processes.

**Methods:** Lungs of C57Bl/6J mice were sliced and treated *ex vivo* with elastase (2.5 μg/ml) or H_2_O_2_ (200 μM) for 16 h. Following treatment, parenchymal structure, airway narrowing, and gene expression levels of alveolar Type I and II cell repair were assessed.

**Results:** Following elastase, but not H_2_O_2_ treatment, slices showed a significant increase in mean linear intercept (Lmi), reflective of emphysema. Only elastase-treated slices showed disorganization of elastin and collagen fibers. In addition, elastase treatment lowered both alveolar Type I and II marker expression, whereas H_2_O_2_ stimulation lowered alveolar Type I marker expression only. Furthermore, elastase-treated slices showed enhanced methacholine-induced airway narrowing as reflected by increased pEC50 (5.87 at basal vs. 6.50 after elastase treatment) and Emax values (47.96 vs. 67.30%), and impaired chloroquine-induced airway opening. The increase in pEC50 correlated with an increase in mean Lmi.

**Conclusion:** Using this model, we show that structural disruption of elastin fibers leads to impaired alveolar repair, disruption of the parenchymal compartment, and altered airway biomechanics, enhancing airway contraction. This finding may have implications for COPD, as the amount of elastin fiber and parenchymal tissue disruption is associated with disease severity. Therefore, we suggest that PCLS can be used to model certain aspects of COPD pathophysiology and that the parenchymal tissue damage observed in COPD contributes to lung function decline by disrupting airway biomechanics. Targeting the parenchymal compartment may therefore be a promising therapeutic target in the treatment of COPD.

## Introduction

Chronic obstructive pulmonary disease (COPD) is a disabling disease and life-threatening. COPD is characterized by an abnormal inflammatory response of the lungs to noxious particles or gases, and this abnormal response is associated with progressive airflow limitation. Long-term exposure to cigarette smoke is a major risk factor for the development of COPD (Pauwels et al., 2001; Rabe et al., 2007). Major pathophysiological characteristics that enhance the decline of lung function and contribute to airway obstruction are inflammation, airway wall remodeling, bronchoconstriction, mucus hypersecretion, high levels of oxidative stress, and an abnormal increase in air spaces (emphysema; Barnes et al., 2003). Worldwide, COPD is the fourth leading cause of death. By 2020 it expected to be the third leading cause of death (COPD, 2016). Available treatments can delay disease progression to some extent, but recovery or normalization of loss of lung function is not possible. Bronchodilators are currently the golden standard for COPD treatment as they reduce bronchoconstriction and airflow obstruction by inducing airway smooth muscle (ASM) relaxation. However, even the most recent combination therapies of long-acting β2 agonists and long-acting anticholinergics have a limited effect on improving lung function since they improve trough FEV1 by a range of ~150–200 mL only (Buhl et al., 2015; Vogelmeier et al., 2013). Although this improvement is clinically significant, clearly other factors than the ASM contribute to the decline of lung function in COPD.

The parenchyma around the airways is known to have an important role in airway narrowing (Bidan et al., 2015). The alveolar tissue is defined by the parenchyma and it consists of a large proportion of the extracellular matrix (ECM) and interstitial cells. The parenchymal compartment is connected to the airways via parenchymal tethers. These tethers transmit forces to the airways, linking chest movements during inspiration to airway opening (Suki and Bates, 2008, 2011; Lauzon et al., 2012). The parenchymal mechanical and structural properties therefore have a major impact on airway mechanics. In healthy lung tissue, the parenchyma maintains the elastic recoil which counteracts airway narrowing. Parenchymal tethers on the outside of an airway transmit trans-pulmonary pressure to the airway wall, thereby opposing the shortening of the ASM (Ma and Bates, 2014). As a result, parenchymal tethering supports the relaxant effect of deep inspiration and reduces bronchoconstriction (An et al., 2007). Changes of parenchymal properties are therefore likely to affect airway narrowing (Bidan et al., 2015). In COPD, the parenchyma and ECM are altered, leading to a loss of elastic recoil (Eurlings et al., 2014; Bidan et al., 2015). These alterations, caused by the presence of elastolytic enzymes and oxidative stress, lead to damage of the parenchyma, and ECM (Chung and Adcock, 2008; Kirkham and Barnes, 2013). Therefore, the increased levels elastolytic enzymes and oxidative likely affect airway mechanics in COPD and enhance bronchoconstriction. In order to have a better understanding of enhanced airway narrowing in COPD, it is therefore important to investigate the role of the parenchymal compartment. This role is especially interesting in mild or moderate COPD, as changes in the parenchymal compartment are not very profound yet in this stage, and possibly still reversible. In order to develop a therapeutic approach to restore parenchymal properties and possibly reduce airway narrowing in COPD, a model in which the underlying pathological processes can be investigated is needed. A previous study by Khan et al. demonstrated that *ex vivo* elastase treatment of mouse lung slices enhanced the velocity of acetylcholine-induced contraction, while the velocity of relaxation was significantly suppressed (Khan et al., 2007). These results indicate that parenchymal damage may indeed enhance airway narrowing.

In the present work, we aimed to expand this model by studying the impact of low level elastase and oxidative stress exposure on airway narrowing and relaxation in relation to parenchymal and ECM structure, and (in)activation of alveolar epithelial repair. We used an *ex vivo* Precision Cut Lung Slice (PCLS) model to mimic these aspects of COPD pathophysiology. PCLS, unlike isolated airway rings, have the advantage that the attachments between the small airways and the surrounding parenchyma remain intact during preparation. Therefore, PCLS are suitable to study the relationship between parenchymal structure and airway function. In addition, the benefit of such an *ex vivo* model is that it enables all the experimental conditions to be performed within the same animal and thus helps limit the number of animals needed and their discomfort as compared to an *in vivo* approach. We hypothesized that *ex vivo* treatment of lung slices with elastase or H_2_O_2_ would damage the parenchyma and therefore enhance bronchoconstriction.

## Materials and methods

### Antibodies and reagents

Methacholine (MCh) was obtained from ICN Biomedicals (Zoetermeer, the Netherlands). Alexa Fluor® 488 Phalloidin was purchased from Life technologies. Mouse anti-E-cadherin was obtained from BD Biosciences (Bedford, MA, USA), and Cy3-conjugated secondary antibody was purchased from Jackson ImmunoResearch (West Grove PA, USA). Elastase from porcine pancreas Type IV and chloroquine were received from Sigma-Aldrich (Zwijndrecht, The Netherlands).

### Animals

C57bl/6 male and female mice (weight 23–41 g; age 12–40 weeks) were obtained from Innoser (Lelystad, The Netherlands). Animals were maintained on mouse chow and tap water *ad libitum* in a humidity- and temperature-controlled room at 24°C with a 12 h light/dark cycle. All experiments were performed according to the national guidelines and upon approval of the experimental procedures by the local Animal Care and Use committee of Groningen University, DEC number 6815A.

### Precision-cut lung slices

Precision-cut lung slices were prepared as described previously (Oenema et al., 2013). Animals were euthanized by subcutaneous injection with ketamine (40 mg/kg, Alfasan, Woerden, The Netherlands) and dexdomitor (0.5 mg/kg, Orion Pharma, Mechelen, Belgium). Following euthanization, the trachea was cannulated, and the animal was ex-sanguinated via the aorta abdominalis. Subsequently, the lungs were inflated through the cannula with a low melting-point agarose solution (1.5% final concentration (Gerbu Biotechnik GmbH, Wieblingen, Germany) in CaCl2 (0.9 mM), MgSO_4_ (0.4 mM), KCl (2.7 mM), NaCl (58.2 mM), NaH_2_PO_4_ (0.6 mM), glucose (8.4 mM), NaHCO_3_ (13 mM), HEPES (12.6 mM), sodium pyruvate (0.5 mM), glutamine (1 mM), MEM-amino acids mixture (1:50), and MEM-vitamins mixture (1:100), pH = 7.2). Following inflation, lungs were placed on ice for 15 min, so that the agarose could solidify for slicing. Next, the lungs were separated into individual lobes. These lobes were used to prepare tissue cores, after which the lobes were sliced at a thickness of 250 μm, which was the same for all further experimental procedures. Slicing was performed in medium composed of CaCl_2_ (1.8 mM), MgSO_4_ (0.8 mM), KCl (5.4 mM), NaCl (116.4 mM), NaH_2_PO_4_ (1.2 mM), glucose (16.7 mM), NaHCO_3_ (26.1 mM), HEPES (25.2 mM), pH = 7.2, using a tissue slicer (CompresstomeTM VF- 300 microtome, Precisionary Instruments, San Jose CA, USA). Lung slices were incubated in a humid atmosphere under 5% CO_2_/95% air at 37°C. Every 30 min slices were washed (four times in total). PCLS were incubated in DMEM supplemented with sodium pyruvate (1 mM), MEM non-essential amino acid mixture (1:100; Gibco® by Life Technologies), gentamycin (45 μg/ml; Gibco® by Life Technologies), penicillin (100 U/ml), streptomycin (100 μg/ml), and amphotericin B (1.5 μg/ml; Gibco® by Life Technologies). Slices were cultured at 37°C in a humidified atmosphere under 5% CO_2_/95% air in 12-well tissue culture plates, using three to four slices per well. Matched slices from the same mouse were treated with elastase (0–2.5 μg/ml, Sigma Aldrich) or H_2_O_2_ (0–800 μM, Merck) for 16 h. Following treatment, slices were washed twice with medium and incubated for another 24 h after which the slices were collected. Previous work from our lab (unpublished) demonstrated that mouse lung slice viability is preserved after 72 h of culturing, as mitochondrial activity did not change. This indicates that the lung slice is viable for at least 3 days. Our experiments were all performed within 56 h after sacrifice.

### mRNA isolation and real-time PCR

Total RNA was extracted from PCLS by using the Maxwell 16 instrument and corresponding Maxwell 16 LEV simply RNA tissue kit (Promega, Madison, USA) for automated purification according to manufacturer's instructions. The Reverse Transcription System (Promega, Madison, USA) was used to reverse transcribe equal amounts of total RNA (1 μg). cDNA was diluted four times after which 1 μl was subjected to real-time PCR This was done with the Illumina Eco Personal QPCR System (Westburg, Leusden, the Netherlands) using FastStart Universal SYBR Green Master (Rox) from Roche Applied Science (Mannheim, Germany). Real-time PCR was performed with denaturation at 95°C for 30 s, annealing at 59°C for 30 s and extension at 72°C for 30 s for 40 cycles followed by 5 min at 72°C. The amount of target gene was normalized to the endogenous reference genes β-2 microglobulin (B2M) and ribosomal protein L13A (RPL13). Genetic markers in treated or untreated slices from the same mouse were incubated in parallel for a similar time interval and were compared and expressed as percent basal. Analysis of RT-PCR data was performed using LinRegPCR analysis software (Ruijter et al., 2009, 2013). Primer sets used to analyze gene expression are shown in Table 1.

**Table 1:** **Primers used for RT-PCR analysis**.

**mRNA**	**Primer**
Mouse B2m	Fwd 5′-ACCGTCTACTGGGATCGAGA-3′
	Rev 5′-TGCTATTTCTTTCTGCGTGCAT-3′
Mouse Rpl13a	Fwd 5′-AGAAGCAGATCTTGAGGTTACGG-3′
	Rev 5′-GTTCACACCAGGAGTCCGTT-3′
Mouse Aqp5	Fwd 5′-CTTGTGGGGATCTACTTCACCG-3′
	Rev 5′-AAGTAGAGGATTGCAGCCAGG-3′
Mouse T1α	Fwd 5′-TCACCCCAATAGAGATGGCTTG-3′
	Rev 5′-GGGCAAGTTGGAAGCTCTCTT-3′
Mouse Rage	Fwd 5′-CACAGGCTCTGTGGGTGAG-3′
	Rev 5′-TTCAGCTCTGCACGTTCCTC-3′
Mouse Con43	Fwd 5′-TCCTTTTCCTTTGACTTCAGCCTC-3′
	Rev 5′-TCTGAAAATGAAGAGCACCGACA-3′
Mouse Sftpc	Fwd 5′-GGAGCACCGGAAACTCAGAA-3′
	Rev 5′-GGAGCCGCTGGTAGTCATAC-3′

### Tissue staining and confocal laser scanning microscopy to visualize parenchymal cells

To visualize the parenchyma of the PCLS, slices were stained for F-actin and E-cadherin. PCLS were fixed for 15 min at 4°C in cytoskeletal buffer (CB) (10 mM Tris base, 150 mM NaCl, 5 mM EGTA, 5 mM MgCl_2_, and 5 mM glucose at pH 6.1) containing 3% paraformaldehyde (PFA). PCLS were then permeabilized by incubation for 5 min at 4°C in CB containing 3% PFA and 0.3% Triton X-100. Subsequently, PCLS were washed twice with 4°C CB. For immunofluorescence microscopy, fixed PCLS were first blocked for 1 h at room temperature in Cyto-TBS buffer (200 mM Tris base, 154 mM NaCl, 20 mM EGTA and 20 mM MgCl_2_at pH 7.2) containing 1% bovine serum albumin and 2% normal donkey serum. PCLS were incubated with primary antibody (E-Cadherin, 1:100, BD biosciences) overnight at 4°C in Cyto-TBS containing 0.1% Tween 20 (Cyto-TBST). The next day, PCLS were incubated with Alexa Fluor® 488 Phalloidin (1:100, Life technologies) and Cy3-conjugated secondary antibody (1:50, Jackson ImmunoResearch) for 2 h at room temperature in Cyto-TBST containing 1% BSA. Between incubation steps slices were washed with Cyto-TBST. Following staining, coverslips were mounted using ProLong Gold antifade reagent (Invitrogen). Fluorescence was determined with a confocal laser scanning microscope (CLSM) equipped with true confocal scanner (TCS; SP8 Leica, Heidelberg, Germany), using a 200x lens. To avoid bleed through, sequential scans were performed. AlexaFluor488 was excited using the 488 nm blue laser line, and Cy™3 was excited using the 552 nm green laser line. All images were recorded in the linear range, at an image resolution of 1024 × 1024 pixels and with a pinhole size of 1 Airy unit, while avoiding local saturation. The images presented here show a single z-scan. ImageJ 1.48d was used to further process images (Schindelin et al., 2012).

### 2-photon imaging and autofluorescence to visualize the ECM

2-Photon and multiphoton excitation fluorescence (MPEF) imaging were used to visualize collagen and elastin polymers, respectively, as described previously (Abraham and Hogg, 2010). Following stimulation with either elastase or H_2_O_2_, PCLS were washed twice with PBS and directly mounted on coverslips. Under excitation at 820 nm, the collagen bundles naturally emitted a second harmonic generation signal collected around 410 nm. Elastin was visualized by using its endogenous fluorescence. Images from elastin were generated by using infrared laser (excitation wavelength 880 nm). The measured broadband emission spectrum ranged from 455 to 650 nm with a peak at ~500 nm.

### Mean linear intercept (Lmi)

To assess emphysema in the PCLS, the mean linear intercept (Lmi) was determined as a measure of mean distance of free airspace, as described previously (van der Strate et al., 2006). Following staining with Alexa Fluor® 488 Phalloidin (1:100, Life technologies) the alveolar structure was visualized by confocal microscopy (magnification 200x). Two fields per animal were used to determine Lmi. As the lungs were filled with agarose under a varying pressure, Lmi differs between animals due to the experimental protocol. However, in this PCLS model animals served as their own control as all experimental conditions were performed within the same animal. Hence, the treatment effect on Lmi was normalized (percent basal) within the animal, and these normalized values were compared between animals. Therefore, the variance in Lmi caused by the experimental protocol was excluded.

### Airway narrowing studies

Airway narrowing studies were performed within the same mouse on untreated slices and on slices treated with elastase (2.5 μg/ml) or H_2_O_2_ (200 μM). Dose response curves for MCh (10^−9^–10^−3^M) were recorded, after which the airways were dilated using the bitter taste receptor agonist chloroquine (10^−3^M, Sigma-Aldrich). A nylon mesh and metal washer were used to fixate the lung slice, as described previously (Rosner et al., 2014). Lung slice images were captured in time-lapse (1 frame per 2 s) using a microscope (Eclipse, TS100; Nikon). To quantify airway luminal area, image acquisition software (NIS-elements; Nikon) was used. Luminal area is expressed as percent basal.

### Data analysis (statistics)

Values reported for all data are represented as mean ± SEM. The statistical significance of differences between means was determined on log transformed data by Student's *t*-test or one-way ANOVA, followed by a Bonferroni correction where appropriate. Log-transformation was performed prior to the statistic calculations when data were normalized to percentage of baseline. While such normalization can be useful to demonstrate an effect of treatment relative to control, it distorts Gaussian distribution of data. Log transformation of is then necessary to re-obtain Gaussian distribution and parametric testing is in such cases allowed. Differences were considered to be statistically significant when *p* < 0.05.

## Results

### Elastase, but not H_2_O_2_ treatment disrupts the parenchyma

**Figure 1:**
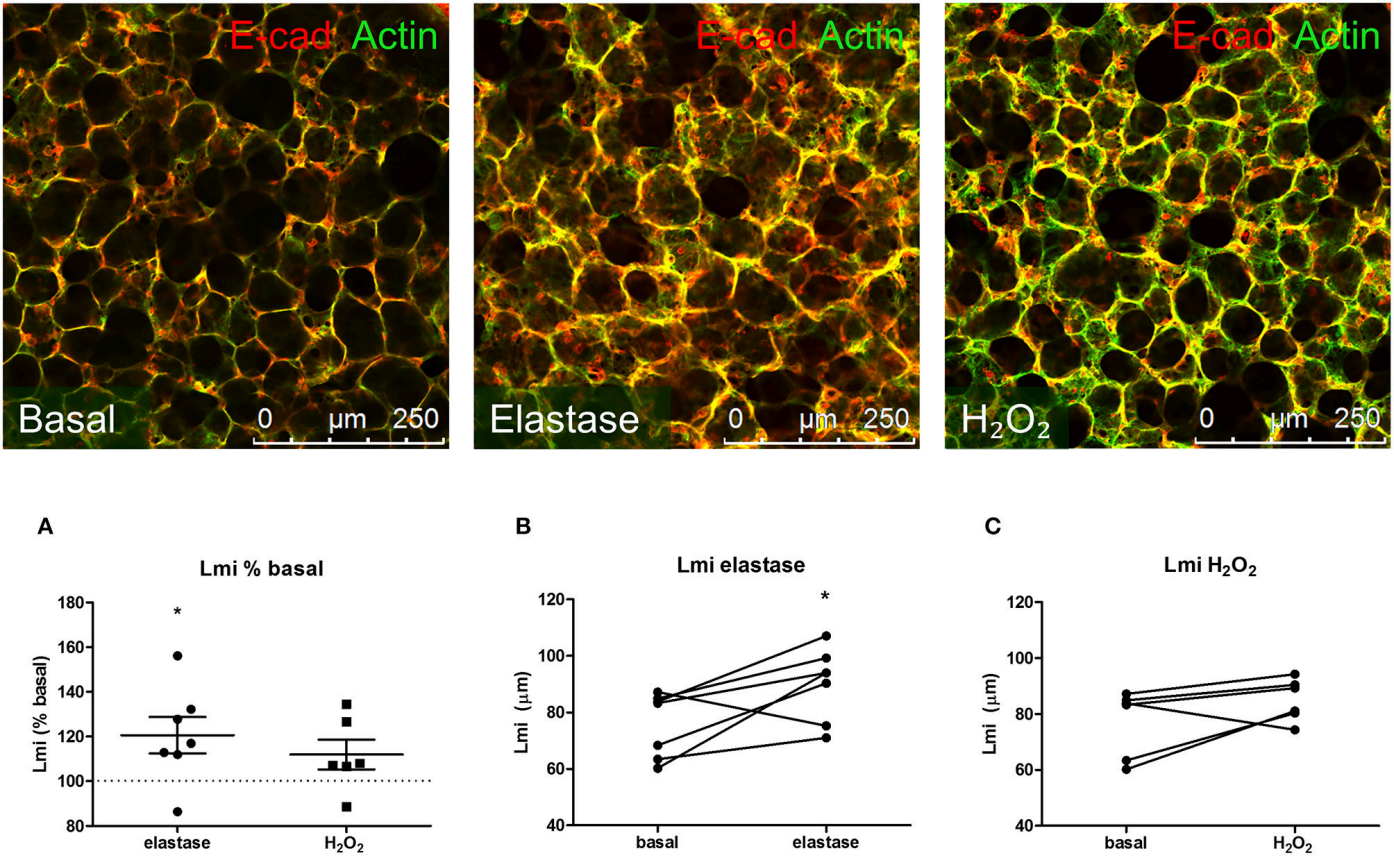
**Elastase, but not H_**2**_O_**2**_ treatment increases Lmi**. PCLS were exposed to elastase (2.5 μg/ml, *n* = 6) or H_2_O_2_ (200 μM, *n* = 5) for 16 h. After stimulation, slices were washed twice with medium and incubated for 24 h in medium. **(A)** Following incubation, slices were stained for F-actin filaments (green) and E-cadherin (red) and Lmi was assessed as % basal. **(B,C)** Lmi following treatments shown in μm. The statistical significance of differences between means was determined on log transformed data by Student's *t*-test **(A)** or by Student's *t*-test **(B,C)**. Data represent mean ± SEM, ^*^p < 0.05 compared to basal control.

**Figure 2:**
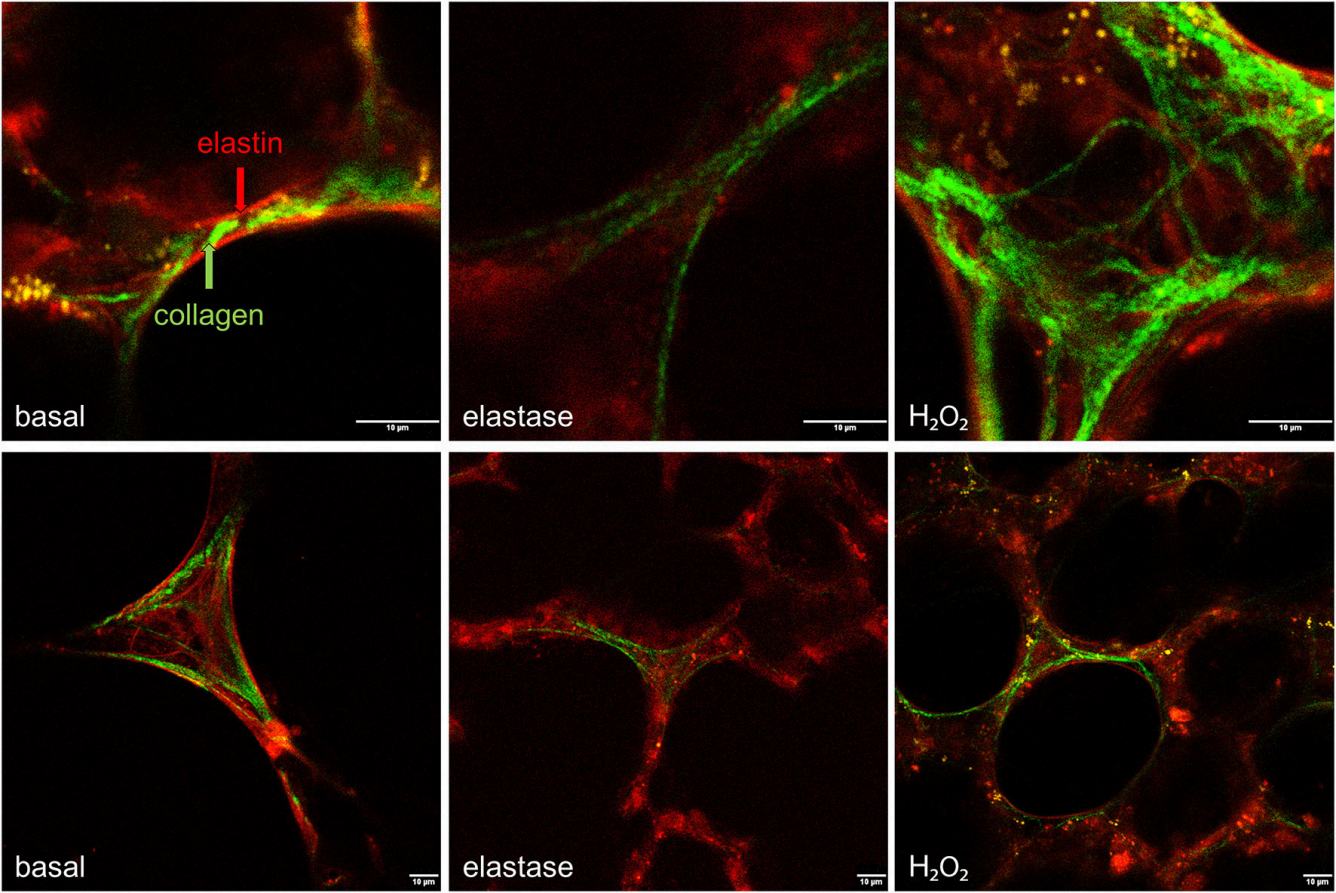
**Elastase, but not H_**2**_O_**2**_ treatment disrupts the structural organization of elastin and collagen**. PCLS were exposed to elastase (2.5 μg/ml) or H_2_O_2_ (200 μM) for 16 h. After stimulation, slices were washed twice with medium and incubated for 24 h in medium. 2-Photon and multiphoton excitation fluorescence (MPEF) imaging were used to visualize collagen and elastin polymers. Following elastase, but not H_2_O_2_, elastin and collagen showed a disrupted fiber organization.

Our first aim was to create tissue damage in the PCLS resembling the tissue damage seen in COPD. Therefore, we first investigated whether elastase and H_2_O_2_ were able to induce parenchymal tissue damage. PCLS were treated with increasing concentrations of elastase (0, 0.16, 0.31, 0.63, 1.25, and 2.5 μg/ml) and H_2_O_2_ (0, 50, 100, 200, 400, 800 μM) and ECM and alveolar epithelial markers were assessed. Based on these initial results (data not shown due to a low n), 2.5 μg/ml elastase and 200 μM of H_2_O_2_ were chosen for all the following experiments. Following elastase treatment gene expression levels of Sftpc decreased (moderately), with little difference between the dosage used. The effect of elastase treatment on ECM marker gene expression levels was rather variable. Confocal images of the parenchymal structure demonstrated that only the highest dose of 2.5 μg/ml elastase enhanced Lmi. Based on these observations, we chose to work with 2.5 μg/ml elastase in further experiments. H_2_O_2_ treatment induced small increases or decreases of Sftpc and ECM markers, depending on the concentration. The highest decrease in Sftpc and ECM markers was observed at the highest dose of 800 μM. However, at this dose we observed visually that the PCLS were very fragile and too disintegrated to use. Therefore, the effect of H_2_O_2_ 800 μM treatment on Sftpc and ECM gene expression levels is more likely to reflect general cell death than a specific effect of H_2_O_2_. Based on these findings we chose to work with 200 μM H_2_O_2_, as these slices showed only small decreases in Sftpc and ECM gene expression levels and were still intact. To assess emphysema in the PCLS, the Lmi was determined as a measure of mean distance of free airspace. Following elastase, but not H_2_O_2_ stimulation, the mean free distance in air spaces was increased as compared to basal (set at 100%) as reflected by an enhanced Lmi [elastase 120.6% (*p* = 0.04) and H_2_O_2_ 111.9% (*p* = 0.76); Figures 1A–C]. 2-Photon and MPEF imaging of elastase-treated slices showed a disrupted elastin and collagen fiber organization, whereas H_2_O_2_-treated slices did not show this effect. Under basal conditions, both elastin and collagen fibers were clearly present. The elastin fibers had a straight appearance whereas the collagen fibers were coiled (Figure 2). Under elastase, but not under H_2_O_2_, conditions, the elastin fibers were absent whereas the collagen fibers became more stretched (Figure 2).

### Elastase and treatment decreases gene expression levels of alveolar type I and II cell markers

**Figure 3:**
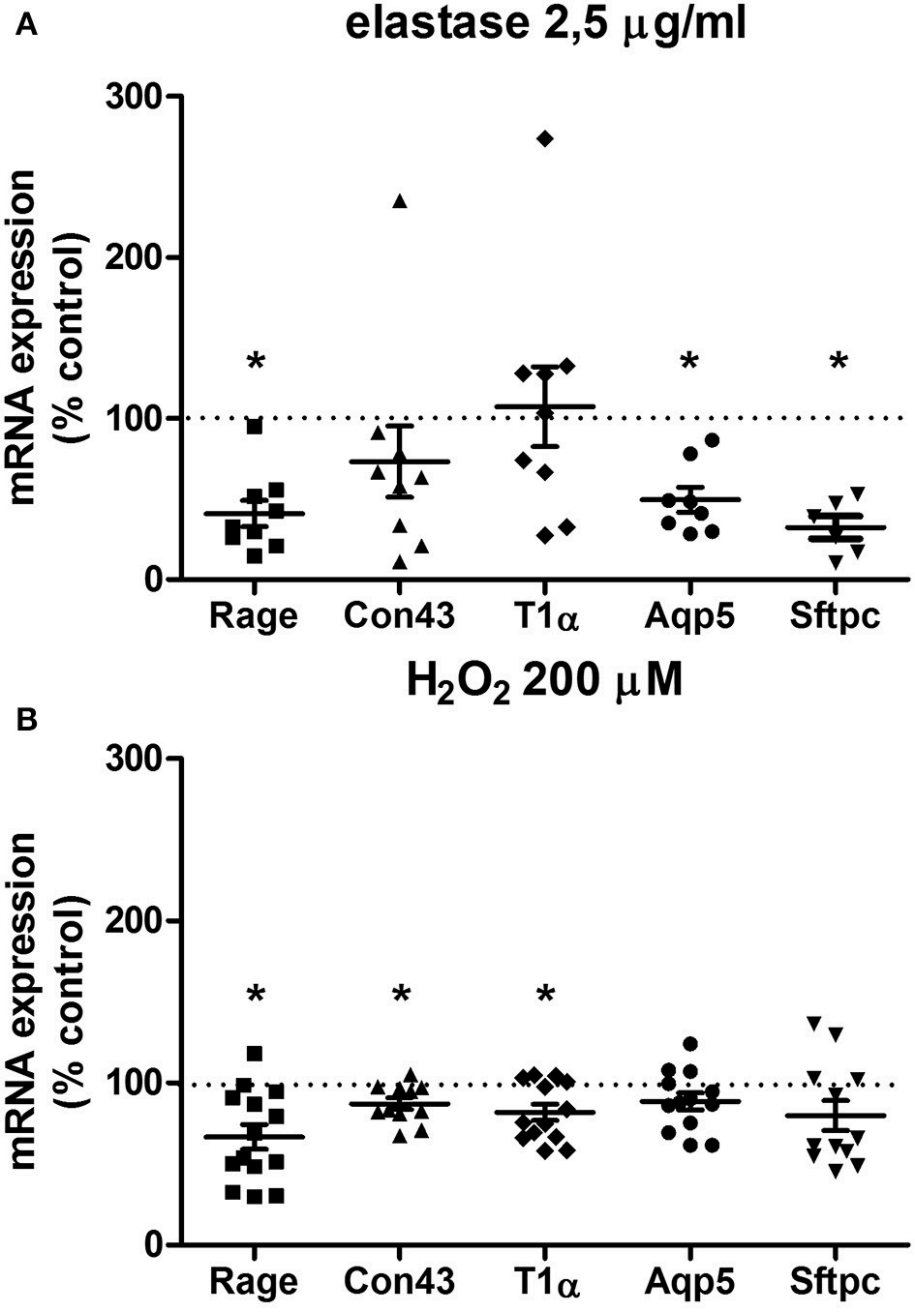
**Elastase and H_**2**_O_**2**_ treatment alter mRNA expression levels of alveolar makers**. PCLS were exposed to **(A)** elastase (2.5 μg/ml, *n* = 9) or **(B)** H_2_O_2_ (200 μM, *n* = 14) for 16 h. After stimulation, slices were washed twice with medium and incubated for 24 h in medium. The statistical significance of differences between means was determined on log transformed data by Student's *t*-test followed by a Bonferroni correction. Data represent mean ± SEM, ^*^p < 0.05 compared to basal control.

To further investigate the parenchymal damage induced by elastase and H_2_O_2,_ markers of alveolar epithelial Type I and II cells were assessed: T1α and Aqp5 (specific for alveolar Type I cells), Con43 and Rage (both alveolar Type I cell associated), and Sftpc (specific for alveolar Type II cells; McElroy and Kasper, 2004; Smirnova et al., 2016). In our model, the structural damage caused by elastase was associated with a specific decrease in expression of alveolar type I and II markers. Following elastase (2.5 μg/ml) stimulation for 16 h, gene expression levels of Aqp5, Rage, and Sftpc, but not Con43 or T1α, were decreased (Figure 3A). H_2_O_2_treatment (200 μM) for 16 h decreased gene expression levels of Rage, Con43, and T1α, but not of Aqp5 or Sftpc (Figure 3B). Taken together, this shows that in addition to disrupting the parenchymal matrix, elastase, but not H_2_O_2_, decreases gene expression levels of alveolar Type II markers.

### Elastase, but not H_2_O_2_ treatment enhances airway narrowing

**Figure 4:**
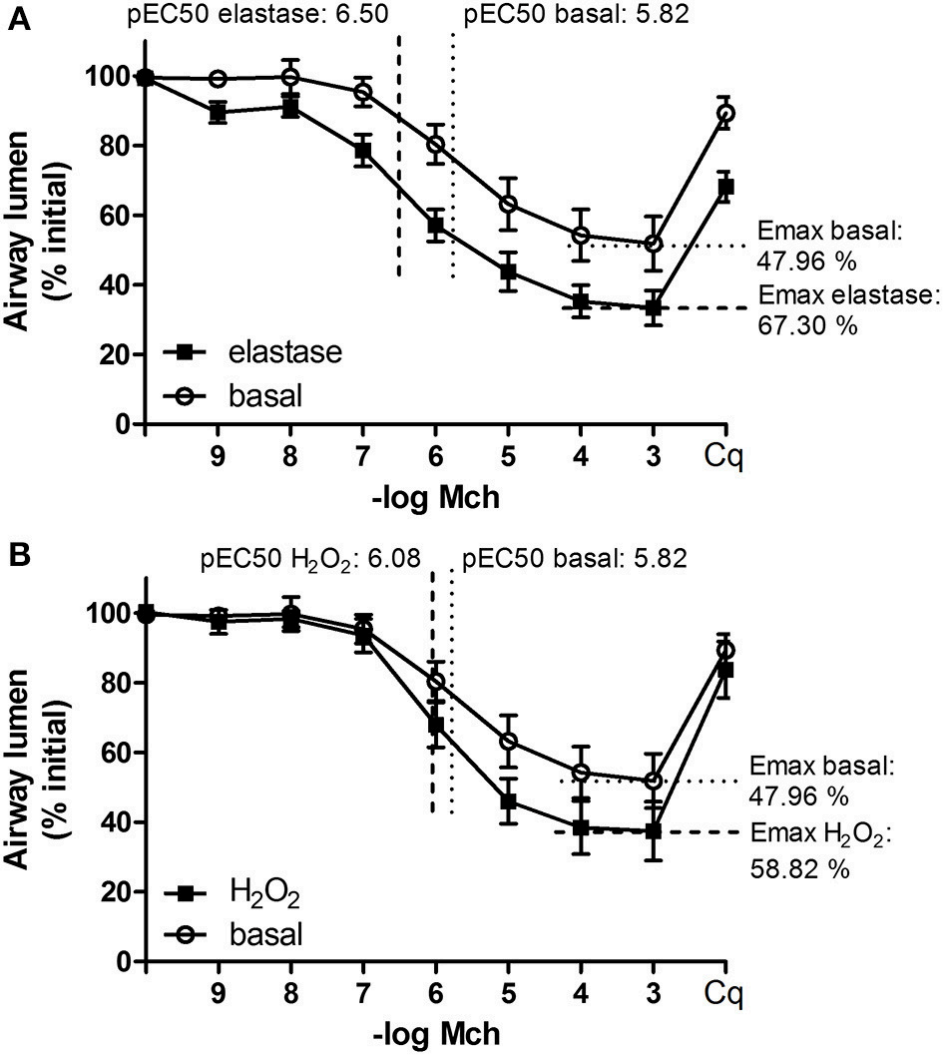
**Elastase, but not H_**2**_O_**2**_ treatment enhances MCh-induced airway narrowing**. PCLS were exposed to elastase (2.5 μg/ml, *n* = 10) or H_2_O_2_ (200 μM, *n* = 10) for 16 h. After stimulation, slices were washed twice with medium and incubated for 24 h in medium. Following incubation, MCh-induced airway narrowing was assessed. Lung slice images were captured in time-lapse (1 frame per 2 s) using an inverted phase contrast microscope (Eclipse, TS100; Nikon). Airway luminal area was quantified using image acquisition software (NIS-elements; Nikon), and expressed as percent basal. **(A)** MCh-induced airway narrowing following elastase treatment. Elastase treatment increased pEC50 values significantly (*p* < 0.05, compared to basal control). **(B)** MCh-induced airway narrowing following H_2_O_2_ treament. The statistical significance of differences between means was determined on log transformed data by one-way ANOVA followed by Bonferonni testing. Data represent mean ± SEM.

**Figure 5:**
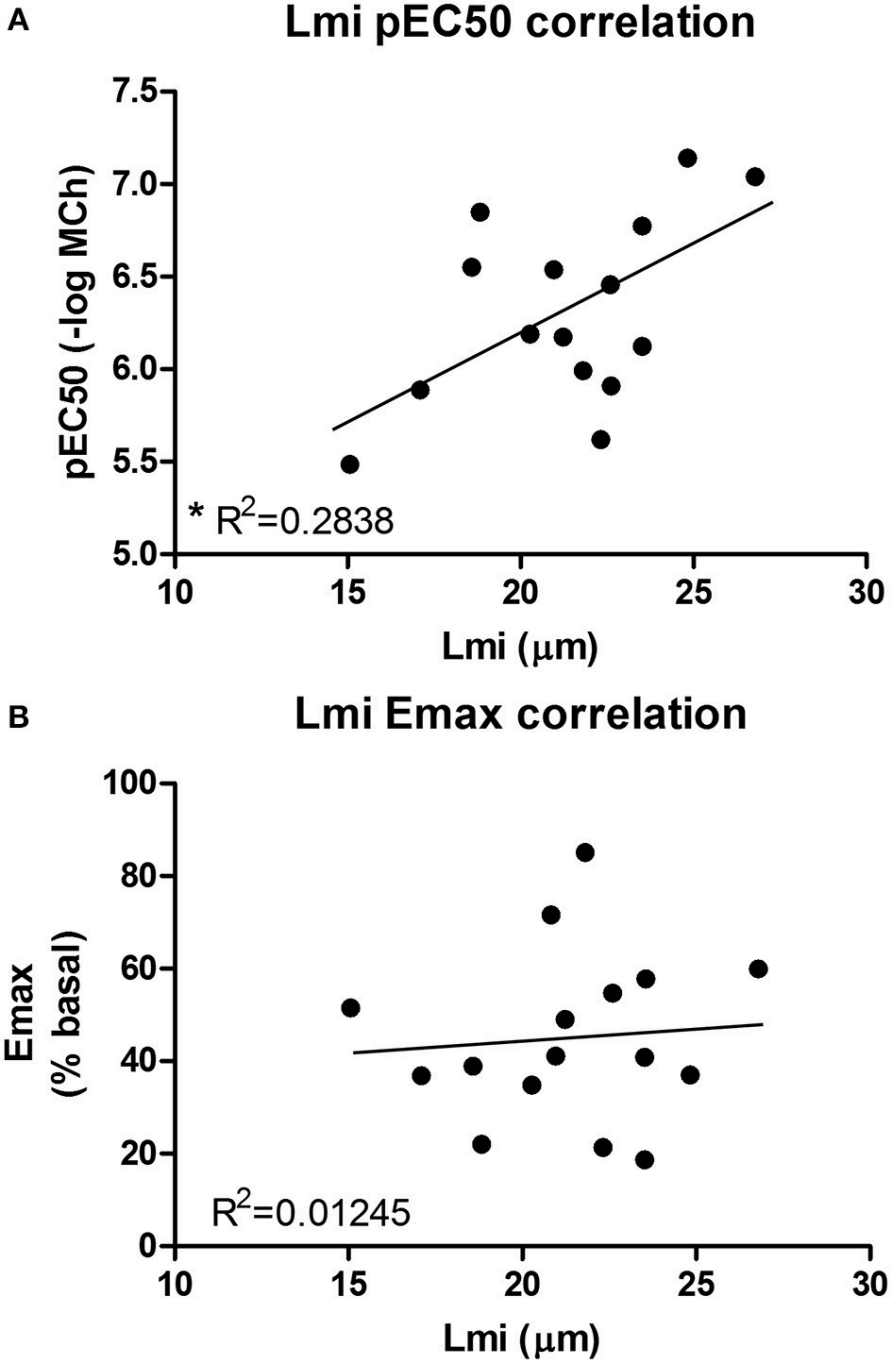
**An increased pEC50 value correlates with an increased Lmi. (A)** Lmi (μm) and pEC50 values for all conditions were combined. It was found that an increased pEC50 value correlates with an increased Lmi (*R*^2^ = 0.2838, *p* < 0.05). **(B)** Emax values did not correlate with Lmi.

**Figure 6:**
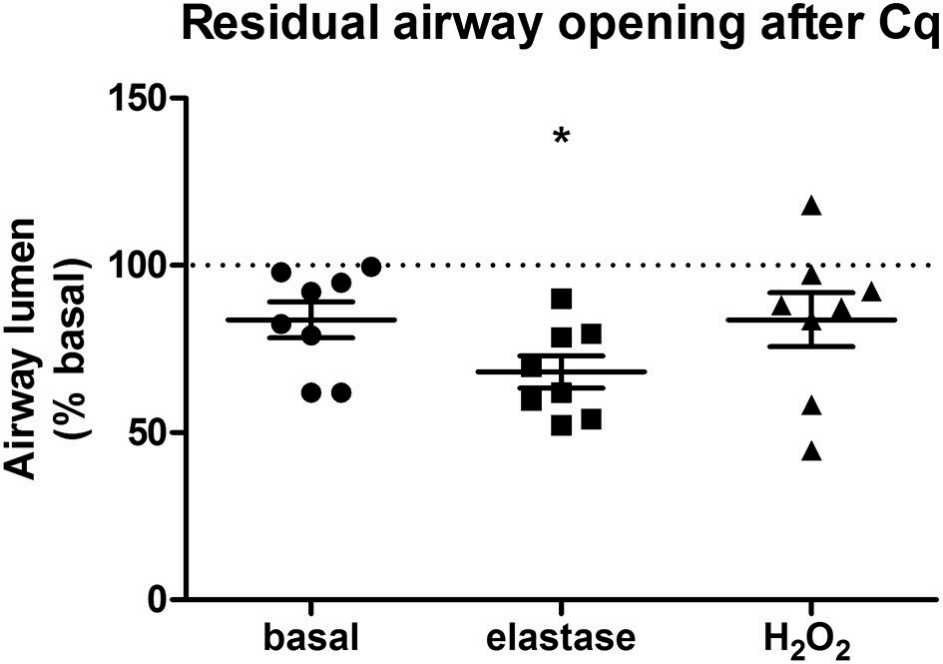
**Elastase, but not H_**2**_O_**2**_ treatment impairs chloroquine-induced airway opening**. PCLS were exposed to elastase (2.5 μg/ml, *n* = 8) or H_2_O_2_ (200 μM, *n* = 8) for 16 h. After stimulation, slices were washed twice with medium and incubated for 24 h in medium. Following incubation, airway narrowing was induced by an increasing dose of MCh. Subsequently, airway relaxation was induced with chloroquine. Elastase, but not H_2_O_2_ treatment, impaired chloroquine-induced relaxation of contracted airways. The statistical significance of differences between means was determined on log transformed data by Student's *t*-test. Data represent mean ± SEM, ^*^p < 0.05 compared to basal control.

Our second aim was to study the relationship between the parenchyma and airway functionality. Therefore, we investigated the effect of elastase and H_2_O_2_ treatment on MCh-induced airway narrowing. Elastase enhanced MCh-induced airway narrowing as shown by a significant increase in pEC50 (5.87 vs. 6.50, *p* < 0.001), and increased Emax (47.96 vs. 67.30% contraction), although not significantly (Figure 4A). H_2_O_2_ did not change pEC50 (5.87 vs. 6.08), nor Emax (47.96 vs. 58.82% contraction (Figure 4B). Interestingly, when values for all conditions were combined, we found pEC50 correlated with Lmi, whereas Emax did not (Figures 5A,B). Subsequent to maximal methacholine-induced airway narrowing, we applied chloroquine as a relaxant to induce airway re-opening. Chloroquine was chosen for this purpose as β_2_-agonists failed to do so in these murine peripheral airways (not shown). Airways in slices treated with elastase did not fully return to their original size after chloroquine-induced relaxation, in contrast to slices treated with H_2_O_2_ (Figure 6). Taken together, these findings indicate that elastase, but not H_2_O_2_ treatment enhances airway narrowing in PCLS and limits airway re-opening to its original size.

## Discussion

COPD is characterized by progressive airflow limitation, and it is increasingly recognized that the parenchymal compartment may play an important role in airway narrowing. Our aim was to set up an *ex vivo* PCLS model mimicking structural abnormalities that resemble COPD and to integrate multiple readouts in order to study their inter-relationship. Tissue damage was established in the PCLS with either elastase or H_2_O_2_. We show that *ex vivo* elastase, but not H_2_O_2,_ treatment leads to disruption of the parenchymal compartment and to enhanced MCh-induced airway narrowing. Elastase-induced damage was reflected by an increased Lmi and a disorganization of elastin and collagen fibers. The structural damage caused by elastase was associated with a specific decrease in both alveolar Type I and II markers (Aqp5, Rage, and Sftpc) mRNA expression. H_2_O_2_ treatment decreased gene expression levels of alveolar Type I cells (Rage, Con43, and T1α) only. As COPD is characterized by both alveolar Type I and II injury, this result indicates that in the PCLS, elastase treatment better reproduces tissue damage seen in COPD than H_2_O_2_ treatment.

We observed a clear relationship between lung structure and airway function. This was demonstrated by the finding that elastase treatment enhanced MCh-induced airway narrowing as reflected by an increased pEC50 and Emax values as compared to control slices. However, a limitation of the PCLS model when studying lung structure and airway biomechanics is the lack of pre-strain or cyclic stretching due to tidal breathing. The relative distribution of forces within the lung has been proposed as a main player in tissue destruction, and hence is a key contributor to disease development (Kononov et al., 2001; Suki and Bates, 2011; Yi et al., 2016). *In vivo* pre-strain and cyclic stretching are likely able to enhance tissue degradation and are therefore likely to influence lung tissue biomechanics. This should be taken into consideration when interpreting the findings of the presented *ex vivo* PCLS model. The enhanced airway narrowing observed in our model is unlikely due to a direct effect of elastase on smooth muscle α-actin or the M_3_ receptor, as elastase did not increase their gene expression levels (data not shown). In addition, an increased pEC50 value correlated with an increased Lmi. Furthermore, elastase treatment impaired chloroquine-induced airway re-opening. Interestingly, it has been shown that in untreated mouse PCLS, chloroquine, but not the β2-agonist salbutamol, is able to maintain efficacy with increasing contraction (Donovan et al., 2014). Therefore, whereas β2-agonists are sensitive to functional antagonism (Lemoine et al., 1992), this is not the case for chloroquine. This indicates that the impaired chloroquine-induced relaxation following elastase treatment is more likely explained by as loss of elastic recoil due to the absence of functional elastin fibers than by functional antagonism. The complete airway re-opening with choloroquine in H_2_O_2_ treated slices supports this. A previous study by Khan et al. demonstrated that *ex vivo* elastase treatment of mouse lung slices enhanced the velocity of acetylcholine-induced contraction while the velocity of relaxation was significantly suppressed (Khan et al., 2007). In their study, airway narrowing studies using acetylcholine (ACh) were performed directly after 16 h elastase incubation period. The finding by Khan et al. that elastase enhances contraction velocity is similar to our observation that elastase enhances airway narrowing after 16 h incubation and additional 24 h washout period. Although unquantified, Khan et al. observed rupture of the parenchymal tethering to the airway at the peak of contraction following elastase treatment. These results fit with our data showing that increased Lmi correlates with increased pEC50 values, and implies that structural damage is the cause of enhanced airway narrowing at both the 16 and 40 h time point. Furthermore, in another study Khan et al. demonstrated that 16 h treatment of PCLS with collagenase increased velocity of ACh-induced airway contraction to a similar extent as elastase, whereas relaxation velocities were affected to a lesser extent (Khan et al., 2010). Together, the findings in the studies by Khan et al. and the present study indicate that parenchymal damage indeed enhances airway narrowing, and that PCLS are a suitable model to study this interaction.

In contrast to elastase, H_2_O_2_ did not alter Lmi, the pEC50 value or chloroquine-induced airway opening. Collectively, the above-mentioned results show that elastase, and to a lesser extent H_2_O_2_, treatment of PCLS induces tissue damage similar to hallmarks of COPD. In addition, these results indicate that parenchymal disruption is linked to an increased sensitivity of the airways to MCh-induced contraction, whereas the maximal contraction is affected by other processes than parenchymal disruption alone. The observation that H_2_O_2_-treatment did not alter the parenchymal ECM structure or airway narrowing confirms our hypothesis that structural disruption of the parenchyma is necessary for altering airway biomechanics. In addition, these results suggest that alveolar Type I cell damage is not immediately involved in altering biomechanics of the airways as H_2_O_2_ lowered expression levels of alveolar Type I markers without affecting airway narrowing. This indicates that damage to the matrix and the parenchyma are primarily responsible. This finding may have implications for COPD, as the amount of parenchymal tissue disruption is associated with the severity of COPD (Deslee et al., 2009; Bidan et al., 2015).

The differential effect of elastase and H_2_O_2_ treatment on the parenchyma and thus airway narrowing might be explained by how these compounds exert their actions. Elastase directly digests elastin whereas H_2_O_2_ exerts its effects via intracellular signaling pathways and oxidative modification of structural proteins and lipids. It is possible that longer H_2_O_2_ stimulation could have produced structural effects, explaining the small effect of oxidative stress on the Lmi. In our model, we applied the stimulation for 16 h and a subsequent wash-out for 24 h before performing the experiments. The wash-out of 24 h was chosen in order to have a therapeutic time-window for future applications of the model as a tool for efficacy monitoring of experimental treatments. A limitation to our model is the period the slices are viable. In previous experiments (data not shown) we demonstrated that airway contractility in PCLS is optimal between 16 and 52 h after slicing. For this reason, and because we wished to maintain the wash-out period of 24 h, we did not attempt to stimulate the PCLS with H_2_O_2_ for a longer time period.

In addition to having differential effects on the parenchyma, H_2_O_2_ and elastase also affected gene expression levels of alveolar Type I and II markers differentially. Type I cells maintain the alveolar structure and gas diffusion toward the alveolar capillaries. Type II cells are the main source of surfactant proteins, in addition to being the progenitor cells of Type I cells. Both cell types are crucial for maintaining alveolar tissue integrity, and the loss of these cells has a direct effect on the whole parenchyma (Morissette et al., 2009). COPD is characterized by a loss of alveolar epithelial type I and II cells and hence alveolar structure integrity (Barnes, 2016). To investigate the alveolar epithelial repair marker expression after elastase or H_2_O_2_ treatment, several markers were chosen: T1α and Aqp5 (specific for alveolar Type I cells), Con43 and Rage (both alveolar Type I cell associated), and Sftpc (specific for alveolar Type II cells; McElroy and Kasper, 2004; Smirnova et al., 2016). Some of these markers such as Aqp5 and Sftpc are decreased in COPD, and this decrease is correlated with a lower lung function (Wang et al., 2007; Zhao et al., 2014). Following injury of alveolar epithelial cells, the level of expression of alveolar markers may change because of an altered regulation or because of the death of cells expressing the marker. Although both treatments caused a decrease in the mRNA expression levels of alveolar type I markers (Rage, Con43, T1α), only elastase treatment reduced the alveolar Type II marker Sftpc. Alveolar Type II cells are secretory cells, which release components of surfactant and ECM (Fehrenbach, 2001). In addition, they also release the extracellular antioxidants Gpx3 and Sod3 (Folz et al., 1997; Burk et al., 2011; Yamada et al., 2012). H_2_O_2_ may not alter Sftpc gene expression levels because of the antioxidant properties of the alveolar Type II cells, thereby explaining the different effect of elastase and H_2_O_2_ on alveolar maker expression levels. A limitation of this study is that we did not assess PCLS reactivity directly following 16 h of treatment. Hence, we cannot exclude the possibility that H_2_O_2_ had an effect on gene expression levels at this earlier time point that was lost following 24 h incubation. However, as we aim to use this model for monitoring experimental treatments in the future we are interested in creating a model with stable parenchymal disruption. The effect of elastase on expression levels of alveolar Type I and II markers may be explained via several mechanisms. First, elastase-induced elastin fragments are known chemokines for macrophages in an adult murine model of emphysema, and elastin fragments can impair alveologenesis (Houghton et al., 2006; Masood et al., 2015). Second, mechanical stimuli are well-known to affect proliferation and differentiation of stem cells (Shah et al., 2014). As elastase disrupts the parenchyma, it reorganizes the distribution of mechanical forces within the tissue and may therefore affect the differentiation of alveolar Type II cells. Together, these mechanisms could explain how elastase is capable of affecting alveolar marker expression levels. Collectively, the above-mentioned results indicate that elastase-treated PCLS serve as a better model to study the specific relationship between parenchymal disruption and airway narrowing than H_2_O_2_-treated PCLS.

COPD is a progressive disease, and currently no treatment is capable of stopping or reversing the progression of lung decline. Novel therapeutic targets are clearly needed, and the parenchyma is a good candidate. Recently, there has been increasing recognition of the potential importance of the parenchymal compartment and its role in lung tissue mechanics in many lung diseases, including COPD (Suki and Bates, 2011; Bidan et al., 2015). In COPD, the parenchyma and ECM are altered (Eurlings et al., 2014; Bidan et al., 2015). The most abundant changes in the ECM are reductions in the expression or functional organization of elastin fiber, leading to a loss of elastic recoil. Alterations in elastin expression are already present in mild to moderate COPD, and seen in both airways and alveoli of COPD patients (Black et al., 2008). Furthermore, this reduction in elastin expression seems to be similar in mild to moderate and severe COPD (Eurlings et al., 2014). Interestingly, alveolar wall elastin fiber structure is altered in patients with severe COPD. Compared to healthy subjects, elastin fibers from COPD patients are significantly less densely packed, unraveled and loose (Deslee et al., 2009). This indicates that, even though elastin expression is similar in both mild to moderate and severe COPD, the disruption of the structural organization of elastin might contribute to the continuous decline of elastic recoil observed in small airways and parenchyma of patients with COPD (Bidan et al., 2015). The same appears to be the case for collagen in COPD. Studies about the total expression levels of collagen in COPD are inconsistent, but it has been observed that collagen fibers are more disorganized in severe COPD as compared to mild to moderate COPD (Tjin et al., 2014). Furthermore, recent studies demonstrate that expression of genes associated with elastogenesis is altered in COPD (Brandsma et al., 2015, 2016). Among the most upregulated genes were fibulin-5 (FBLN5), elastin (ELN), latent transforming growth factor β binding protein 2 (LTBP2), and microfibrillar associated protein 4 (MFAP4), which are all implicated in elastogenesis. In addition to elevated gene expression levels of FBLN5 this study demonstrated that cleaved, possibly non-functional FBLN5 protein was present in COPD lung tissue, indicating an impaired repair response. Targeting these elastogenesis pathways in COPD may therefore represent a novel therapeutic target.

Disorganization of elastin and a changed organization of collagen fibers were also observed in our PCLS model following treatment with elastase, demonstrating that elastase treatment mimics the damage seen in COPD lung tissue. Under basal conditions, both elastin and collagen fibers were present. Following elastase treatment, the elastin fibers were lost and the collagen fibers became more stretched. The importance of ECM fiber structure is demonstrated by the finding that tissue strips from elastase-treated mice failed at ~50% less stress than control animals, even though the treated animals had a 50% increase in total collagen content of the lung (Ito et al., 2005). These results agree with our finding that collagen fibers become more stretched following elastase treatment; if collagen is already stretched maximally, it will break at a lower deformation compared to control. Moreover, due to structural remodeling, the yield stress (breaking point) of collagen is lower in the emphysematous lung (Suki and Bates, 2008). A lower yield stress of collagen also means a lower resistance to airway contraction, explaining why structural remodeling of collagen can lead to an enhanced airway narrowing, even though total collagen expression levels are increased and Lmi is unchanged. Taken together, these reported findings show that the ECM in the airway and parenchymal compartments of COPD patients is altered as compared to non-COPD controls. One of the most evident alterations is the changed expression level and disorganization of elastin and collagen fibers. In our PCLS model, elastase treatment increased the Lmi, disrupted the fiber organization of elastin and collagen, and enhanced methacholine-induced airway narrowing as reflected by an increased pEC50 value. Importantly, increased Lmi values correlated with increased pEC50 values. Furthermore, elastase treatment impaired chloroquine-induced airway re-opening. Taken together, the above-mentioned findings stress the importance of the contribution of the parenchymal ECM structure to airway narrowing. It is likely that the enhanced bronchoconstriction in COPD is at least partly caused by the parenchymal tissue damage. This role of the parenchymal compartment in airway narrowing is especially interesting in mild or moderate COPD, as changes in the parenchymal compartment are not very profound yet in stage, and possibly still reversible. Therefore, the parenchyma represents a promising target in the treatment of COPD.

In summary, these results demonstrate that PCLS can be used to model structural defects in COPD in a comprehensive manner. To our knowledge, this is the first study integrating multiple read-out parameters such as the parenchyma, epithelial repair marker expression and airway function in one model. Using this model, we show that structural disruption of the parenchymal compartment leads to altered biomechanics of the airways, enhancing airway contraction. This finding may have implications for COPD, as the amount of parenchymal tissue disruption is associated with the severity of the disease. Therefore, we suggest that the parenchymal tissue damage observed in COPD contributes to lung function decline by disrupting airway biomechanics. Targeting the parenchymal compartment may therefore be a promising therapeutic target in the treatment of COPD.

## Author contributions

EV, CB, and RG contributed to the conception or design of the work. EM, SC, and MM contributed to the acquisition and analysis of data. All authors contributed to the interpretation of data. All authors drafted the work or revised it critically for important intellectual content. All authors approved the final version of the manuscript.

## Funding

We would like to thank the Netherlands Organization for Scientific Research and the Netherlands Lung Foundation for financial support (Vidi grant: 016.116.309 and 6.1.14.009, respectively).

### Conflict of interest statement

The authors declare that the research was conducted in the absence of any commercial or financial relationships that could be construed as a potential conflict of interest.
